# Mast Cells Are Identified in the Lung Parenchyma of Wild Mice, Which Can Be Recapitulated in Naturalized Laboratory Mice

**DOI:** 10.3389/fimmu.2021.736692

**Published:** 2021-09-27

**Authors:** Yu-Wen Yeh, Arka Sen Chaudhuri, Ling Zhou, Yu Fang, Preben Boysen, Zou Xiang

**Affiliations:** ^1^ Department of Health Technology and Informatics, Faculty of Health and Social Sciences, The Hong Kong Polytechnic University, Hong Kong, Hong Kong, SAR, China; ^2^ Center for Clinical Laboratory, Affiliated Hospital of Guizhou Medical University, Guiyang, China; ^3^ School for Clinical Laboratory, Guizhou Medical University, Guiyang, China; ^4^ Faculty of Veterinary Medicine, Norwegian University of Life Sciences (NMBU), Oslo, Norway; ^5^ The Hong Kong Polytechnic University Shenzhen Research Institute, Shenzhen, China

**Keywords:** laboratory mouse, mast cell, wild mouse, lung, mouse naturalization

## Abstract

**Background:**

It is well documented that laboratory mice bred and maintained in ultra-hygienic specific pathogen-free (SPF) barriers display reduced richness and complexity of microbiota compared with wild mice. The laboratory mice profoundly lack lung parenchymal mast cells. Hence, we aimed to investigate the lung distribution of mast cells in free-living wild mice.

**Methods:**

Wild house mice were trapped in South-Eastern Norway and Hemtabad, West Bengal, India. C57BL/6 laboratory mice were bred in a purposefully built, closed environment with bedding material obtained from the natural environment in order to normalize the gut microbiota of these laboratory mice to that of the wild mice, and the offspring were collected for study at eight weeks of age.

**Results:**

Mast cells were easily identified at a substantial density in the lung parenchymal tissues of wild mice from both Norway and India, which stands in clear contrast to the rare distribution of lung parenchymal mast cells in the conventional laboratory SPF mice. Consistently, wild mice also expressed higher pulmonary levels of stem cell factor, a critical growth factor for mast cell survival. Higher levels of histamine were recorded in the lung tissues of the wild mice. Interestingly, “naturalized” C57BL/6 laboratory mice which spent their entire life in a semi-natural environment developed lung parenchymal mast cells at an appreciable density.

**Conclusion:**

Our observations support that environmental factors, possibly through modulation of microbiota, may impact the tissue distribution of mast cells in mouse lung parenchyma.

## Introduction

Because of their anatomical and physiological similarity to humans, as well as a number of other advantages, such as the ease of maintenance and breeding in a laboratory setting, house mice (*Mus musculus*) have long been the model species of choice to mimic human diseases in experimental studies (1). However, limitations of translating mouse research in areas where humans are possibly different from mice are also evident. There is a growing concern that laboratory mice do not truthfully mirror relevant aspects of human physiology and pathology, including immune responses, and hence it is quite often difficult to extrapolate results derived from mouse studies to human treatments (2). Lack of precision in data extrapolation from mice to humans may be due to their fundamental divergence at the genetic level over a long evolutionary history. Alternatively, this may also reflect a more recent artefact arising from the creation and breeding of laboratory mouse strains (1). Accumulating experimental evidence suggests that traditional laboratory mice that are bred and maintained in an ultra-hygienic specific pathogen-free (SPF) barrier display reduced richness and complexity of microbiota compared with wild house mice. Discrepant immunological features have been well documented between SPF laboratory mice and wild-caught mice (3–7). Wild populations of house mice are predicted to be a more relevant model to reflect human immune responses (8).

Asthma, a chronic disease characterized by airway inflammation and respiratory symptoms, is one of the most prevalent human diseases affecting the quality of life of more than 300 million people across the globe (9). There is an urgent need for improved treatment plans to tackle this chronic disease. Animal models are critical for elucidating the immunological mechanisms in asthma and developing therapeutic strategies. To date, the established mouse asthma models have relied on the use of laboratory-adapted, inbred strains of mice (e.g., C57BL/6 and BALB/c). Of particular relevance to allergy and asthma, these laboratory strains profoundly lack lung parenchymal mast cells (10), which stands in clear contrast to the presence of abundant mast cell populations in human lungs (11). This observation challenges the relevance of mouse asthma models for understanding human asthma (12).

Mast cells have been described as one of the major types of cells that are involved in the development of asthma and allergy by virtue of their potential to secrete a variety of allergic mediators (13). Mast cells are derived from hematopoietic progenitors in the bone marrow and these progenitors migrate to the vascularized tissues where they further differentiate into mature mast cells. Mast cells are enriched in the skin, around blood vessels, and in mucosal membranes such as the respiratory and gastrointestinal tracts. Tissue-specific distribution of mast cells is dependent on various mediators. It has been shown that after allergen-mediated sensitization in the respiratory tract, CCL2 is locally produced and recruits mast cell progenitors which express CCR2 (14). Stem cell factor (SCF) is one of the major growth and differentiation factors for mast cells (15). In addition to SCF, mast cell growth and differentiation can be facilitated by several other cytokines including IL-3 (16). Tissue mast cells are capable of further differentiating both phenotypically and functionally as a consequence of tissue-specific stimulation under defined microenvironmental conditions. For example, inflamed human lungs are reported to have more tryptase/chymase-producing mast cells compared with non-inflamed lung tissue in which tryptase-producing mast cells are dominant (17, 18). The number of mast cells is increased at sites of allergic inflammation, and there is a correlation between mast cell density in the tissue and the severity of allergic symptoms (19). In allergy, plurivalent antigens bind and crosslink IgE molecules bound to the high-affinity IgE-receptor (FcεRI) expressed on mast cells, resulting in cell degranulation and release of proinflammatory mediator molecules.

Given the literature reports revealing the discrepancies between conventional laboratory mice and wild mice with respect to various immunological features (20), we were intrigued to investigate lung mast cell distribution in free-living wild mice. We captured a number of free-living mice near or in farm-houses in South-Eastern Norway and Hemtabad, West Bengal, India. We examined mast cell distribution in these wild mice and observed substantial numbers of mast cells in the lung parenchyma of these wild mice. All the laboratory control mice, either C57BL/6 or BALB/c, examined in parallel, lacked mast cells in lung parenchyma. We also show that laboratory mice born and raised in a semi-natural environment for rodents could develop lung parenchymal mast cells. Our aim was to understand mast cell biology in relation to environmental and genetic influences, and potentially to improve the mouse model for asthma and allergy research.

## Materials and Methods

### Live-Trapping of Wild Mice and the Source of Laboratory Mice

Wild house mice were caught using our previously described practical methodology on mouse capture (6). Free-living mice were captured in Hemtabad, West Bengal, India. Furthermore, wild-caught and control laboratory mice from Norway were obtained from a previously reported material (6, 21). The *Mus musculus* identity of the wild mice were confirmed as previously described (22) or by PCR genotyping based on the *Mus musculus*-specific GAPDH gene sequences using two sets of primers (forward 5’-TGGCCGGATACCTAGTTCCA-3’; reverse 5’-AGGTGAATCAGGGAAGCAGC-3’, and forward 5’- AACAACTGGCTTTCCACCCA -3’; reverse 5’-ACTGCCTGGTAAAGGTCACG-3’). The specific pathogen-free (SPF) C57BL/6 and BALB/c laboratory mouse controls were obtained from Charles River/Scanbur, Norway, Huafukang Bioscience, Beijing, or bred in-house at the Centralized Animal Facilities at the Hong Kong Polytechnic University. The animal protocols were approved by the Ethics Committee of the Guizhou Medical University. Animal material from Norway was collected under approval from the Norwegian Food Safety Authority (FOTS ID 4788, 6801, 8080 and 8198) and the Norwegian Environment Agency (ref. 2012/693 and 2014/7215).

### Housing and Breeding of Laboratory Mice in a Semi-Natural Environment

Both female and male C57BL/6 mice were housed in mouse pens designed with a naturalistic farm-like environment as previously reported (21), however, without the direct presence of wild-caught mice. Briefly, a purposefully built, closed environment was prepared with bedding material regularly brought in from domestic animal farmhouses, as well as sawdust, soil, compost, twigs and hay. This would recapture a common habitat for the free-living house mouse, aiming to normalize the C57BL/6 mice in terms of the gut microbiota and cellular immunology. Mice were allowed to breed in this closed environment and their progeny were collected for study at 8 weeks of age.

### Formalin-Fixed Paraffin-Embedded Lung Tissue Block Preparation

The lung tissues were fixed in 10% neutral buffered formalin for 24 h at room temperature followed by embedding in paraffin wax. The lung sections were taken from lung lobes avoiding the central airways. Tissue processing was carried out using a Thermo Scientific Excelsior AS Tissue Processor. Consecutive 5-µm sections were generated from each of the FFPE tissue blocks using a standard microtome blade and the sections were fixed onto glass microscope slides.

### Microscopic Examination

Deparaffinized FFPE sections were rehydrated using xylene and downgraded concentrations of alcohol. The slides were stained with 0.1% toluidine blue (Sigma; 89640-5G) for 30 sec, followed by rinsing in 96% ethanol for a few seconds and then dehydrating in absolute ethanol. For tryptase, c-Kit and histamine staining by immunohistochemistry, slides were stained with a rabbit monoclonal antibody against mast cell tryptase (Huabio; ET1610-64), a rat monoclonal antibody against c-Kit (Biolegend; 105822), or rabbit polyclonal antibody against histamine (Abcam; ab37088), followed by staining with a rabbit IgG-specific (Abcam; ab64261) or a rat IgG-specific (Cell Signaling Technology; 7077S) HRP-linked secondary antibody, using the HRP/DAB Detection IHC kit (Abcam; ab64261) according to the manufacturer’s instruction. For H&E staining, slides were stained with hematoxylin (Thermo Scientific; 72711) and eosin (Pioneer Research Chemicals; PRC/66/1) using a standard methodology. Slides were mounted with LAMB DPX mounting medium (Thermo Scientific), and microscopic images were acquired using a Nikon Eclipse Ci-L upright clinical microscope or using a Nikon Ti2-E wide-field microscope. Mast cell density was determined by counting the number of positively stained cells in high power fields (400 ×) per mm^2^. For counting the mast cell, we focused on the lung parenchymal tissues and tried to avoid the area close to the bronchi. For quantitative analysis of histamine expression, optical density (OD) was obtained and processed using ImageJ. Histamine levels were expressed as OD per area. Depending on the density of the cells identified or the frequency of the positive staining, the whole sectioned tissues (toluidine blue), or three (histamine) or five (tryptase and c-Kit) randomly selected high power fields were scanned for enumeration or quantification, or otherwise as indicated.

### Real-Time Quantitative Reverse Transcription PCR

All the FFPE lung blocks and 5-μm sections were stored at room temperature until RNA extraction. Following deparaffinization, total RNA was extracted and purified using an RNeasy FFPE Kit (Qiagen; 73504) according to the manufacturer’s instruction. cDNA was synthesized using the RevertAid First Strand cDNA Synthesis Kit (Thermo Fisher; K1622). qRT-PCR was performed with the ViiA 7 Real-Time PCR System (Applied Biosystems™) using the Power SYBR™ Green PCR Master Mix (Thermo Fisher; A25776). PCR was carried out with an initial incubation at 50°C for 2 min, and 95°C for 2 min, followed by 40 cycles of 95°C for 15 sec and 60°C for 1 min. The specificity of the reaction was verified by melt curve analysis. The relative expression values of each gene were normalized to GAPDH expression and were calculated by the 2-ΔΔCT method. The PCR primer sequences are displayed in **Table S1**.

### Statistical Analysis

A Mann-Whitney *U* test was used to calculate statistical differences between the two comparisons. Where applicable, data are expressed as median with individual mouse data points shown. Outliers were removed based on Grubbs’ test.

## Results

### Wild Mice Exhibit Lung Parenchymal Mast Cells

**Figure 1:**
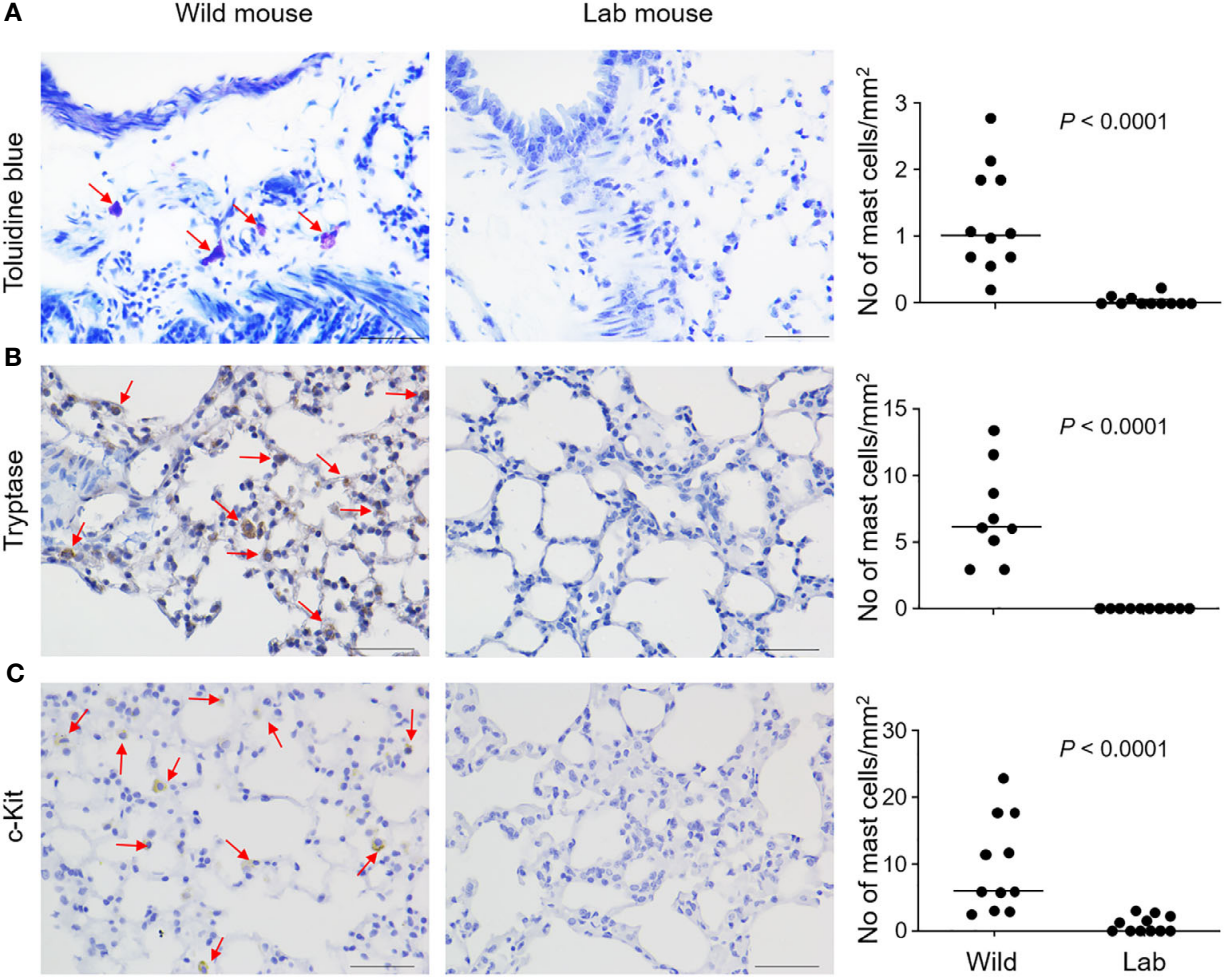
Mast cells are identified in the lung parenchyma of wild free-living mice. Free-living wild mice were trapped at Hemtabad, India (*n* = 11). Their lung tissues, together with those from C57BL/6 laboratory (lab) mice (*n* = 11), were processed and sectioned, followed by staining with the mast cell-specific dye toluidine blue **(A)**, or peroxidase-based immunostaining using an anti-mouse tryptase antibody **(B)** or an anti-mouse c-Kit antibody **(C)**. Arrows indicate mast cells which were stained purple (for toluidine blue) or brown (for immunostaining). Mast cell density was quantified and shown as number of cells per unit area (right panels). Each dot represents an individual mouse and horizontal lines indicate the median. Scale bar: 50 μm. Two wild mice and one lab mouse, which had extremely high values were removed (**B**, right panel) as outliers based on Grubbs’ test (*P* < 0.05).

We trapped free-living wild mice in South-Eastern Norway and Hemtabad, West Bengal, India. Mouse lungs were embedded in paraffin and sectioned for microscopy. Mast cells could be easily identified in the lung parenchyma of wild mice from India according to staining with toluidine blue, a dye that stains mast cell metachromatic granule content, which stands in clear contrast to the rare distribution of lung parenchymal mast cells in the conventional C57BL/6 laboratory mice (**Figure 1A**). Statistically significantly different distributions of lung parenchymal mast cells were observed between wild mice and control laboratory mice (**Figure 1A**). Furthermore, identification of mast cells based on staining for mast cell tryptase (**Figure 1B**) or expression of c-Kit (**Figure 1C**) also supported increased distribution of mast cells in wild mice. A similar trend towards increased lung mast cell numbers was noted in mice trapped in Norway, based on toluidine blue staining (**Figure S1A**) and tryptase expression (**Figure S1B**), although the number of animals was too low to validate statistically. We noted that tryptase and c-Kit staining seemed to be more sensitive than toluidine blue for identifying lung mast cells in wild mice obtained in India. In contrast, tryptase and toluidine blue staining seemed to be consistent for staining lung mast cells in wild mice obtained in Norway. Differential staining sensitivity of toluidine blue for mast cells in different tissues or different species has been observed previously. For example, guinea pig lung mast cells could not be clearly revealed by toluidine blue [(23); and personal communication with Dr Mikael Adner, Stockholm]. Furthermore, we could find more densely populated mast cells around the bronchi in both the laboratory mice and the wild mice (**Figure S2**), which is consistent with a previous study (10). Therefore, in our cell number quantification, we tried to focus on mast cells in the lung parenchyma and avoid the areas around bronchi. The average density of lung parenchymal mast cells in C57BL/6 mice, which was minimal, is also consistent with this previous report (10).

### Wild Mouse Lungs Express Higher Levels of Histamine

**Figure 2:**
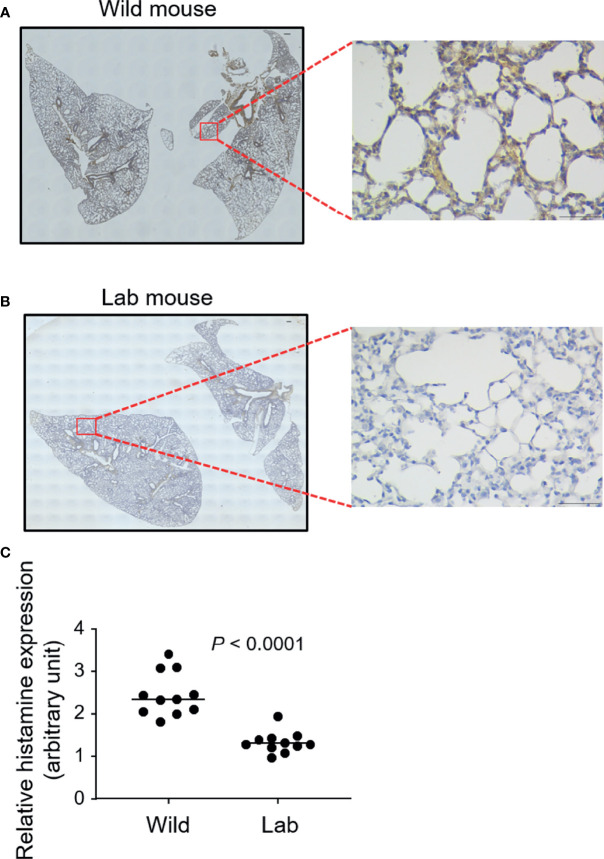
Wild mouse lungs express higher levels of histamine. Free-living wild mice were trapped at Hemtabad, India (*n* = 11) and their lung tissues **(A)**, together with laboratory (lab) mouse (C57BL/6) controls (*n* = 11) **(B)**, were processed and sectioned, followed by peroxidase-based immunostaining using an antihistamine antibody. Magnified views of the highlighted regions are shown. At least three randomly chosen areas were scanned for determining the intensity of histamine which was expressed as optical density (OD) per area **(C)**. Each dot represents an individual mouse and horizontal lines indicate the median. Scale bar: 200 μm or 50 μm (magnified view).

We next examined the lung tissue histamine levels, which correlates with mast cell distribution (24), using immunostaining. Wild mouse lungs were observed to express higher levels of histamine, as confirmed directly from the visual comparison of the microscopic images (**Figures 2A, B**) and based on the staining intensity quantification (**Figure 2C**).

### Wild Mice Express Higher Levels of SCF and Have Similar Overall Lung Tissue Histology as Laboratory Mice

**Figure 3:**
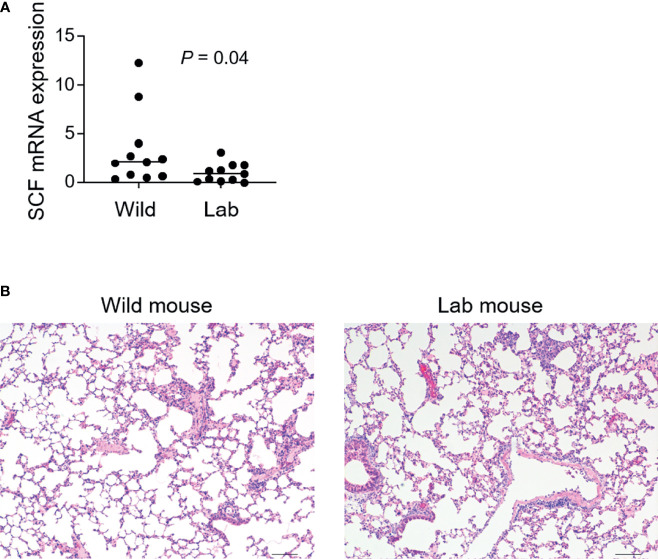
Higher levels of SCF are identified in wild mouse lungs which show similar overall histological features as laboratory mice. Free-living wild mice were trapped at Hemtabad, India. **(A)** RNA was purified from formalin-fixed paraffin-embedded (FFPE) lung tissues of both wild mice (*n* = 11) and C57BL/6 laboratory (lab) mice (*n* = 11), followed by cDNA synthesis. Levels of mRNA expression of SCF were assessed using quantitative reverse transcription PCR. SCF expression was normalized relative to the expression of GAPDH. Each dot represents an individual mouse and horizontal lines indicate the median. **(B)** Lung tissues from wild mice together with lab control mice were processed and sectioned for H&E staining. Shown are the representative images. Scale bar: 100 μm.

Next, we investigated factors underlying the emergence of lung mast cells in the wild mice. We measured a group of cytokines and molecules that are involved in mast cell differentiation and migration, which included SCF, IL-3, IL-4, IL-6, IL-9, TGF-β, VCAM-1, CXCR2 and CCL2 (25, 26). Except for SCF, which demonstrated a modest enhancement in the wild mice (**Figure 3A**), none of the others were found to be enhanced at appreciable levels (data not shown). It has been reported that enriched microbiota can upregulate the production of SCF (27). To investigate whether wild mice developed enhanced lung inflammation as a result of persistent exposure to various types of microbes including pathogens, we compared the overall lung histological features between the wild mice and laboratory mice. Lung inflammation has been reported to be an inducer for recruitment of pulmonary mast cells (28). However, no obvious differences were observed between the laboratory mice and the wild mice trapped in India using H&E staining (**Figure 3B**).

### Laboratory Mice Born and Raised in a Semi-Natural Environment Develop Lung Parenchymal Mast Cells

**Figure 4:**
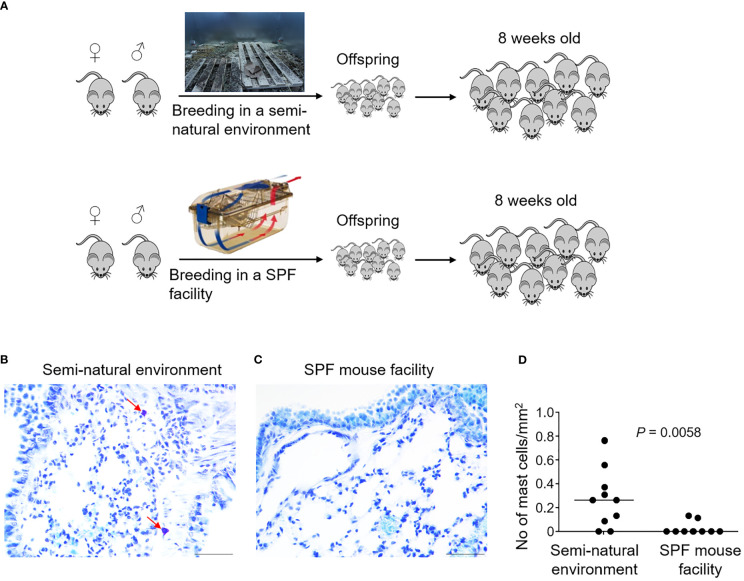
Laboratory mice born and raised in a semi-natural environment develop lung parenchymal mast cells. C57BL/6 laboratory mice were either bred in a purposefully built, closed environment with bedding material from the natural environment at Oslo, Norway, or bred in a conventional specific pathogen-free (SPF) animal facility as indicated **(A)**. Lung tissues were collected from the mice born and raised in the semi-natural environment [**(B)**, *n* = 10] or the SPF facility [**(C)**, *n* = 10] at 8 weeks old. Lung tissue sections were stained with the mast cell-specific dye toluidine blue. Arrows indicate mast cells which were stained purple. Mast cell density was quantified by enumerating toluidine blue-positive mast cells per unit area **(D)**. Each dot represents an individual mouse and horizontal lines indicate the median. Scale bar: 50 μm. One mouse from the SPF group which had an extremely high value was removed as an outlier based on Grubbs’ test (*P* < 0.05).

It is generally assumed that immunological phenotypic adaptation can arise owing to environmental impacts by living in a dirty, natural environment (20). We therefore explored whether it was possible to repopulate lung mast cells in the laboratory mice by exposing them to a natural environment mimicking the natural habitat of wild mice. Laboratory mice were bred in a closed area with bedding material obtained from the natural rodent living environment (**Figure 4A**) (21). We collected offspring mice who spent their entire life in this semi-natural, dirty environment when they were eight weeks old for analysis. Interestingly, these mice showed a significantly higher density of lung parenchymal mast cells compared to the barrier facility-bred and -reared mice as confirmed using toluidine blue staining (**Figures 4B–D**) as well as tryptase staining (**Figure S3**), thus demonstrating that exposure to the natural living environment early in life was associated with the recruitment of mast cells in the lung parenchymal tissues.

## Discussion

We have provided evidence showing that wild mice contained substantially greater numbers of lung parenchymal mast cells compared with the commonly used laboratory mice, which almost completely lacked lung parenchymal mast cells. Our aim was to understand mast cell biology in relation to environmental and genetic influences, and potentially to provide implication for refining relevant mouse models whereby the function of lung mast cells is crucial, e.g., relevant models for asthma research.

**Figure 5:**
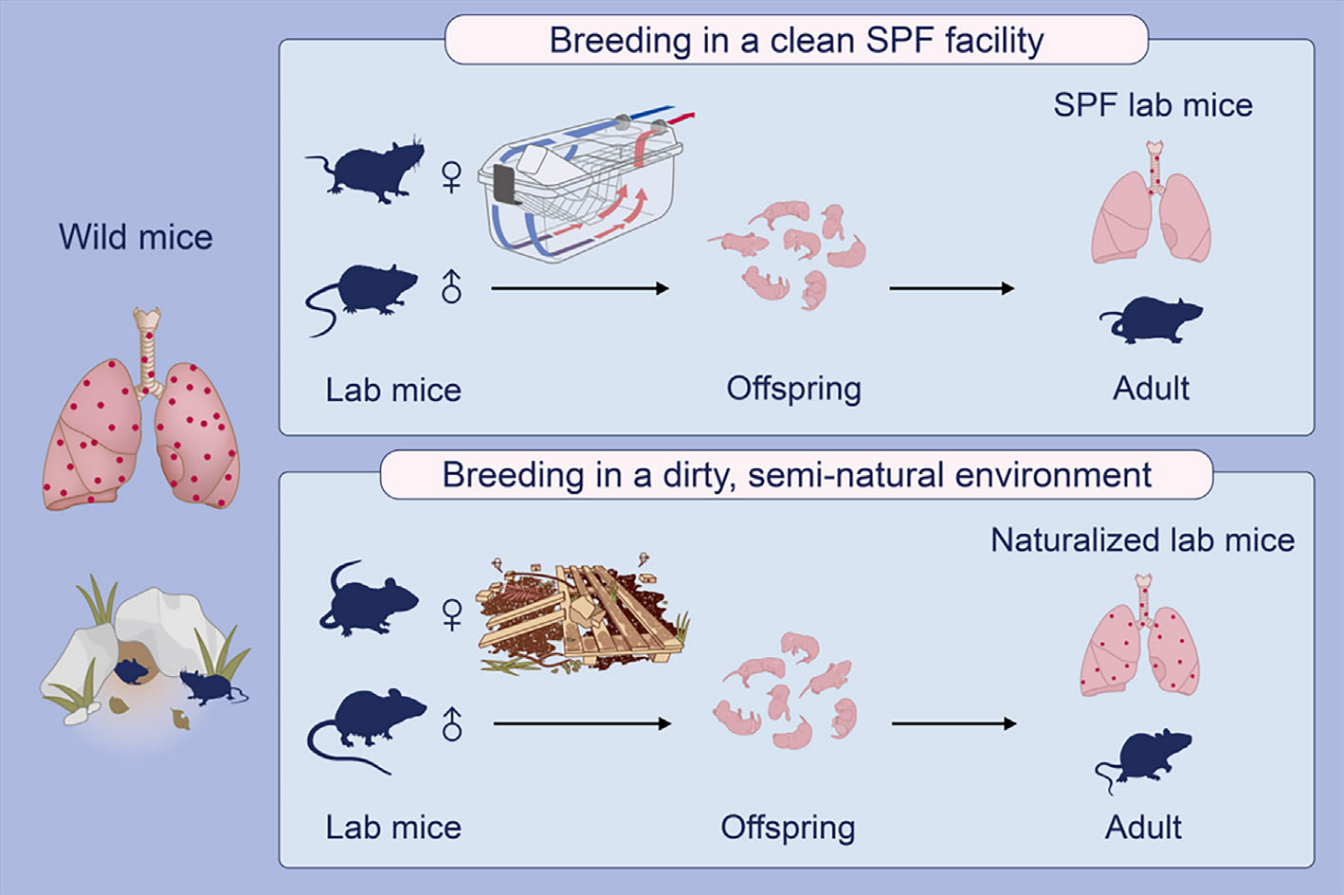
A graphical representation explains a possible environmental impact on the development of lung parenchymal mast cells in mice. Laboratory (lab) mice maintained in ultra-hygienic, specific pathogen-free (SPF) conditions profoundly lack lung parenchymal mast cells, in contrast to the rich presence of mast cells in human lungs (not depicted). Interestingly, free-living wild house mice are found to express lung parenchymal mast cells. Support for an environmental impact on the development of lung mast cells in these mice comes from the fact that mast cells do appear in the lung tissues of ‘naturalized’ lab mice bred in a semi-natural environment with farm-derived bedding materials.

Compared with the abundant expression of mast cells in human lungs, absence of lung parenchymal mast cells in conventional laboratory mice may arise from both genetic and environmental factors. In evolutionary terms, mice and humans diverged between 80 and 90 million years ago (29). An ever growing body of evidence also indicates that gut microbiota can effectively contribute to the shaping of the immune signatures of individuals including mice (30). Therefore, humans and mice, harboring quite different microorganisms, could have developed unique features and functionality of their respective immune system. Our data showing the presence of lung parenchymal mast cells in the wild-caught house mice, to some lesser extent in naturalized laboratory mice, but not in SPF laboratory mice, argues for a strong environmental impact on mast cell tissue distribution (**Figure 5**).

Free-living wild mice inhabit environments that are drastically different from laboratory SPF mice, and mice from these two populations have evolved to demonstrate profoundly different patterns of microbiota (31). We have previously shown that co-housing of SPF laboratory mice with wild mice leads to substantial changes in the fecal microbiota of the laboratory strain after 8-12 weeks (21, 32). Co-housing of laboratory mice with pet store mice can convert their immune phenotypes from originally reflecting human neonates to bearing immune signatures of adult humans (4). Providing laboratory mice with a history of infections that mice normally encounter in the wild changes their blood immune signatures akin to wild mice and adult humans (33). A seminal study showed that breeding laboratory mouse progeny in a wild surrogate mother generates so-called ‘wildling’ mice with a purely inbred genotype but with the microbiota and many of the immune phenotypes of wild mice (34). Recently, wildling mice were shown to develop stronger asthmatic inflammation compared with SPF mice (bioRxiv preprint doi: https://doi.org/10.1101/2021.03.28.437143). Mast cell numbers in the lungs were not reported in these studies, and it would be of interest to investigate if these may be altered in wildlings, which would provide an explanation to their stronger asthmatic responses, and strengthen the link between enriched microbiota and mast cell presence.

Since laboratory mice normally harbor few lung mast cells and poorly respond in asthma models, various approaches have been employed to induce pulmonary recruitment of mast cells in mice. Adjuvant-free sensitization with ovalbumin (OVA) followed by chronic intranasal OVA challenge leads to the recruitment of mast cells in the lung tissue (35). Sensitization with OVA admixed to alum followed by exposing mice to daily challenges with aerosolized OVA for one week (36), intranasal OVA challenges twice weekly for at least one month (37), or three consecutive daily intratracheal OVA challenges (38) induces lung recruitment of mast cells. In the model presented here, sensitization to natural allergens could have similarly recruited mast cells to the lung parenchyma, as an alternative or additional causative factor for our findings. The mode of exposure in naturalized and wild mice may arguably be more in accord with exposures in humans compared to repeated OVA/alum aerosols, but how these models might comparatively play out in translational studies remains to be investigated.

Mice have served and will continue to serve as a valuable research tool for the study of immunology including mast cell biology. Indeed, research based on the use of mouse models have contributed substantially to our knowledge in understanding the roles of mast cells in asthma and allergy. However, preclinical research using animal models that are different from human immunology may account for the discrepancies between predictions of animal models and clinical trial outcomes. From a translational medicine point of view, establishing clinically relevant mouse asthma models may make preclinical research extrapolatable, which can avoid waste of time and research resources. Indeed, the majority of asthma drugs that pass preclinical testing never survive clinical trials. Among the reasons for the high failure rate of drug development, limitations of appropriate animal models used for drug testing obviously constitute a major one (1). Our naturalization approach may provide an alternative practical solution to the establishment of mouse models with resident lung mast cells.

## Data Availability Statement

The original contributions presented in the study are included in the article/**Supplementary Material**. Further inquiries can be directed to the corresponding authors.

## Ethics Statement

The animal study was reviewed and approved by Ethics Committee of the Guizhou Medical University.

## Author Contributions

YF, PB, and ZX conceived and designed the study. Y-WY, AC, and LZ performed research. All authors contributed to data analysis and interpretation. Y-WY, PB, and ZX wrote the paper. All authors contributed to the article and approved the submitted version.

## Funding

This work was supported by grants from Shenzhen Science and Technology Commission, China (JCYJ20170818103812122) (ZX), General research fund from Hong Kong Research Grants Council (15104418) (ZX), and the National Natural Science Foundation of China (81560266 and 81760294) (YF).

## Conflict of Interest

The authors declare that the research was conducted in the absence of any commercial or financial relationships that could be construed as a potential conflict of interest.

## Publisher’s Note

All claims expressed in this article are solely those of the authors and do not necessarily represent those of their affiliated organizations, or those of the publisher, the editors and the reviewers. Any product that may be evaluated in this article, or claim that may be made by its manufacturer, is not guaranteed or endorsed by the publisher.
