# Supplementary material for: Mast Cells Are Identified in the Lung Parenchyma of Wild Mice, Which Can Be Recapitulated in Naturalized Laboratory Mice
Source: Front Immunol. 2021 Sep 27;12:736692. doi: 10.3389/fimmu.2021.736692 (PMC8502827; doi:10.3389/fimmu.2021.736692)
Supplement: Supplementary file 1 [file DataSheet_1.pdf]

Mast cells are identified in the lung parenchyma of wild mice which can be recapitulated in naturalized laboratory mice

Yu-Wen Yeh, Arka Sen Chaudhuri, Ling Zhou, Yu Fang, Preben Boysen, Zou Xiang

Supplementary documents

**TABLE S1.** List of the primer sequences used for real-time quantitative reverse transcription PCR (qRT-PCR)

| Gene name | Gene sequence (5'- 3') |
|---|---|
| GAPDH forward | GGTGAAGGTCGGTGTGAACGGA |
| GAPDH reverse | TGTTAGTGGGGTCTCGCTCCTG |
| SCF forward | CAATTACAAGCGAAATGAGAGCC |
| SCF reverse | CCCTGAAGACTCGGGCCTA |
| IL-3 forward | CTGCCTACATCTGCGAATGACT |
| IL-3 reverse | CAGATCGTTAAGGTGGACCATG |
| IL-4 forward | ACAGGAGAAGGGACGCCAT |
| IL-4 reverse | GAAGCCCTACAGACGAGCTCA |
| IL-6 forward | CCACTTCACAAGTCGGAGGCTTA |
| IL-6 reverse | CCAGTTTGGTAGCATCCATCATTTC |
| IL-9 forward | GTGACATACATCCTTGCCTC |
| IL-9 reverse | GTGGTACAATCATCAGTTGGG |
| TGF-β forward | CGCTGAATCGAAAGCCCTGTA |
| TGF-β reverse | CGCTGAATCGAAAGCCCTGTA |
| VAMC-1 forward | TGAACCCAAACAGAGGCAGAGT |
| VAMC-1 reverse | GGTATCCCATCACTTGAGCAGG |
| CXCR2 forward | ATGCCCTCTATTCTGCCAGAT |
| CXCR2 reverse | GTGCTCCGGTTGTATAAGATGAC |
| CCL2 forward | GTTGGCTCAGCCAGATGCA |
| CCL2 reverse | AGCCTACTCATTGGGATCATCTTG |

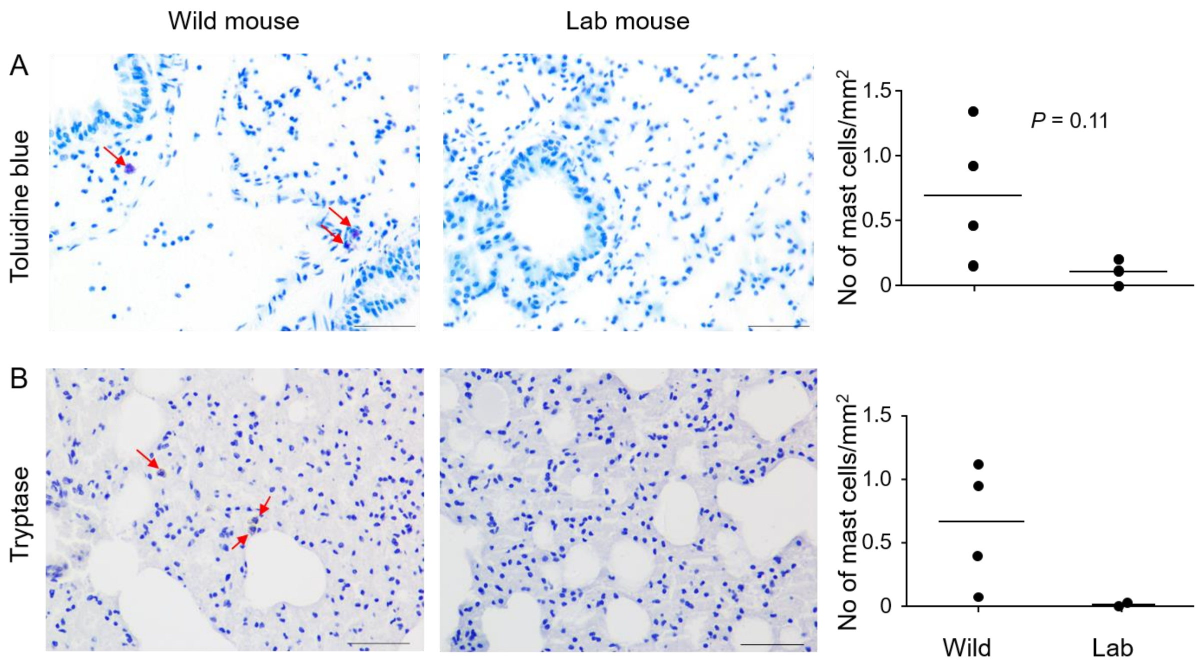


**FIGURE S1.** Mast cells are identified in the lung parenchyma of wild free-living mice from Norway. Free-living wild mice were trapped at South-Eastern Norway ($n$ = 4). Their lung tissues, together with those from locally matched C57BL/6 laboratory (lab) mice ($n$ = 3), were processed and sectioned, followed by staining with the mast cell-specific dye toluidine blue (A), or peroxidase-based immunostaining using an anti-mouse tryptase antibody (B). Arrows indicate mast cells which were stained purple (for toluidine blue) or brown (for tryptase immunostaining). The whole sections were scanned for quantifying mast cell density which is shown as number of cells per unit area (right panels). Each dot represents an individual mouse and horizontal lines indicate the median. Scale bar: 50 µm. One lab mouse was removed because of an unreasonably high value (B, right column).

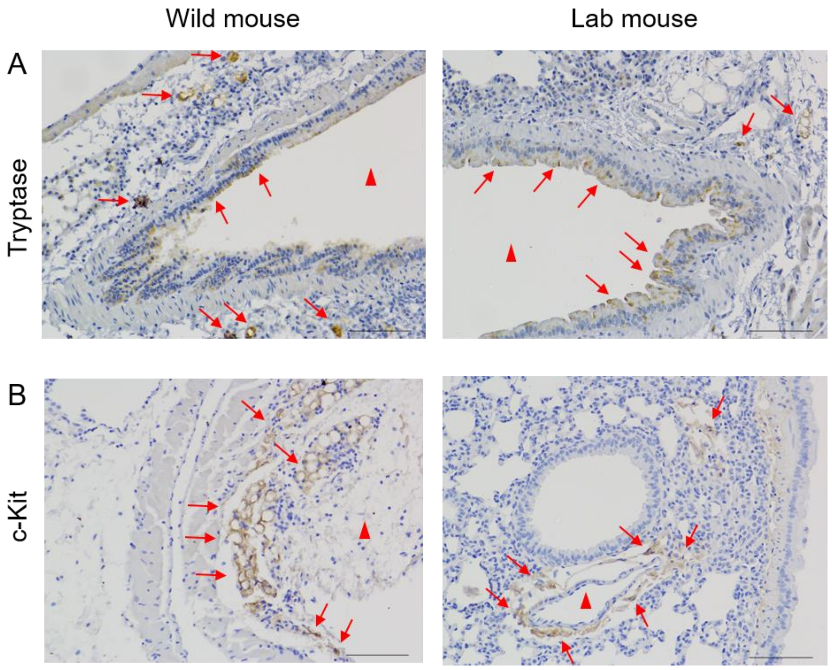


**FIGURE S2.** Mast cells are more frequently distributed around bronchi. Free-living wild mice were trapped at Hemtabad, India. Their lung tissues, together with those from C57BL/6 laboratory (lab) mice, were processed and sectioned, followed by peroxidase-based immunostaining using an anti-mouse tryptase antibody (A) or an anti-mouse c-Kit antibody (B). Arrows indicate individual mast cells or regions with mast cells which were stained brown. Arrow heads indicate bronchi. Representative microscopic pictures are shown. Scale bar: 100 μm.

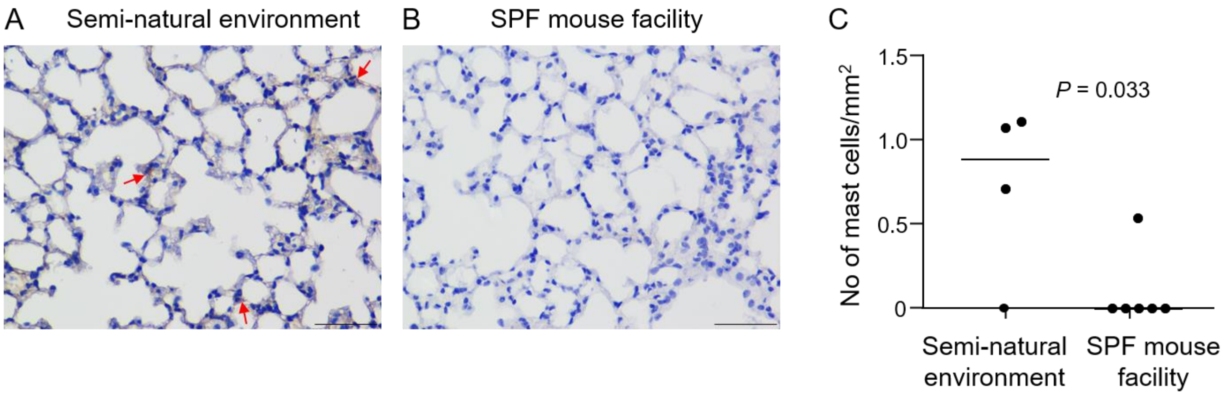


**FIGURE S3.** Laboratory mice born and raised in a semi-natural environment develop lung parenchymal mast cells. C57BL/6 laboratory mice were either bred in a semi-natural environment (*n* = 4) (A) or in a conventional specific pathogen-free (SPF) animal facility (*n* = 6) (B) as described in Figure 4A. Lung tissues were processed for peroxidase-based immunostaining using an anti-mouse tryptase antibody. Mast cell density was quantified by enumerating tryptase-positive mast cells per unit area (C). Arrows indicate tryptase-containing mast cells. Each dot represents an individual mouse and horizontal lines indicate the median. Scale bar: 50 µm.
